# Electrically programmable solid-state metasurfaces via flash localised heating

**DOI:** 10.1038/s41377-023-01078-6

**Published:** 2023-02-22

**Authors:** Khosro Zangeneh Kamali, Lei Xu, Nikita Gagrani, Hark Hoe Tan, Chennupati Jagadish, Andrey Miroshnichenko, Dragomir Neshev, Mohsen Rahmani

**Affiliations:** 1 ARC Centre of Excellence for Transformative Meta-Optical Systems (TMOS), Research School of Physics, The Australian National University, Canberra, ACT 2601 Australia; 2 Advanced Optics and Photonics Laboratory, Department of Engineering, School of Science and Technology, Nottingham Trent University, Nottingham, NG11 8NS UK; 3 School of Engineering and Information Technology, University of New South Wales, Canberra, ACT 2600 Australia

**Keywords:** Nanoparticles, Imaging and sensing

## Abstract

In the last decades, metasurfaces have attracted much attention because of their extraordinary light-scattering properties. However, their inherently static geometry is an obstacle to many applications where dynamic tunability in their optical behaviour is required. Currently, there is a quest to enable dynamic tuning of metasurface properties, particularly with fast tuning rate, large modulation by small electrical signals, solid state and programmable across multiple pixels. Here, we demonstrate electrically tunable metasurfaces driven by thermo-optic effect and flash-heating in silicon. We show a 9-fold change in transmission by <5 V biasing voltage and the modulation rise-time of <625 µs. Our device consists of a silicon hole array metasurface encapsulated by transparent conducting oxide as a localised heater. It allows for video frame rate optical switching over multiple pixels that can be electrically programmed. Some of the advantages of the proposed tuning method compared with other methods are the possibility to apply it for modulation in the visible and near-infrared region, large modulation depth, working at transmission regime, exhibiting low optical loss, low input voltage requirement, and operating with higher than video-rate switching speed. The device is furthermore compatible with modern electronic display technologies and could be ideal for personal electronic devices such as flat displays, virtual reality holography and light detection and ranging, where fast, solid-state and transparent optical switches are required.

## Abstract (web summary)

A rapid and programmable amplitude modulator based on the thermo-optical effect has been demonstrated by integrating transparent microheaters with metasurfaces.

## Graphical abstract

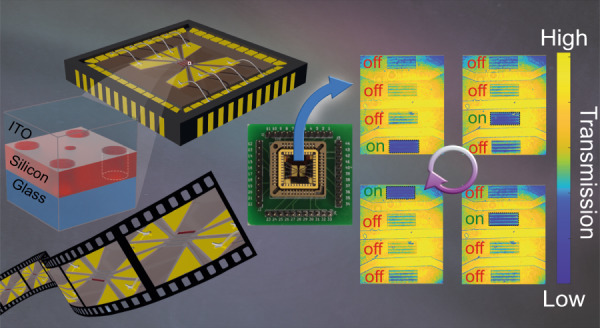

## Introduction

Metasurfaces have recently realised many revolutionary applications, such as metalenses, equation solvers, beam shapers and holographic projections^1–5^. Electrically driven metasurfaces are at the centre of attention in the metasurface community due to their integrability with personal electronic devices. However, to date, none of the electrical tuning approaches can simultaneously enable fast, large, transparent, solid-state and polarisation-independent modulations. Carrier injection methods show ultra-fast modulation response at near-infrared or longer wavelengths, but the modulation contrast is weak and inherently induces large absorption in the system^6–12^. Embedding metasurfaces within liquid crystals provide stronger modulation, but the modulation speed is limited, the cells are not solid-state, the components are bulky, and they induce polarisation effects^13,14^. Electro-optically tunable metasurfaces, based on the Pockels effect, are fast but require materials with inversion asymmetry and the modulation depth is often small^15–20^. Electrically driven phase-change material (PCM) metasurfaces offer good opportunities, however, reverting their phase from crystalline to amorphous state and back often requires challenging treatments or introduces strong absorption in the visible and near-infrared (IR) spectral range^21–24^. More importantly, this process requires high-temperature treatments (>600 °C). Such high temperatures may permanently degrade the CMOS and semiconductor devices, result in humongous noise readings, and change their performance. As a result, PCM metasurfaces are not compatible to be integrated with CMOS devices. Overall, there is still a quest for a novel platform that meets the requirements for electrical tunability in metasurfaces. More detailed information about different electrically tunable metasurfaces can be found in Supplementary Section 1.

Here, we introduce and experimentally demonstrate an electrically tunable CMOS-compatible technique that simultaneously addresses the limitations of various tuning techniques. Our platform exploits silicon’s large thermo-optical effect and provides an order of magnitude faster modulation than video frame rates, polarisation-independent operation, miniaturised building block and fully solid-state components. Our metasurfaces are controlled via electrically driven localised transparent heaters that switch the metasurface optical properties by biased voltages <5 V. By applying an asymmetric driving voltage, we achieve flash heating, leading to 625 μs modulation time. It is worth mentioning that such a modulation time is more than 10-fold faster than the detection limit of the human eye (13 ms)^25^. Therefore, despite the operational temperature of ~200 °C, it can still be integrated with CMOS devices. Such a performance makes our metasurface the first integrable metasurface within cutting-edge gadgets, where a fast, solid-state and transparent optical switch is required, such as flat displays, virtual reality dynamic holography, or light detection and ranging (LIDAR). We demonstrate the first generation of such devices, where the tuning parameters can be further improved by optimising microheater dimensions (see Supplementary Information, Section 2), input voltage profile, and cooling approaches.

## Results

### Hole array metasurface

**Fig. 1:**
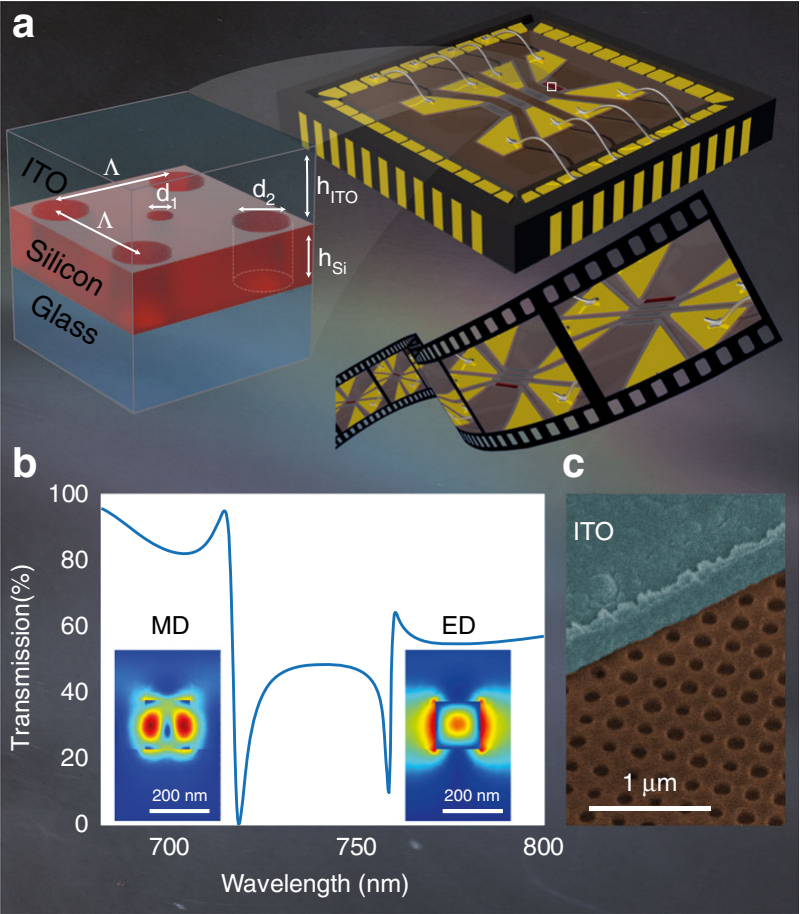
**Electrically switchable metasurface pixels by flash localised heating. a** Illustration of the fabricated sample, composed of designed nanoholes in a silicon film, so-called hole array metasurfaces, embedded in an ITO transparent heater. The pixel’s optical responses are switched by applying a voltage across the contacts, which locally heats the metasurfaces. The thermo-optical effect results in an alteration of the metasurface scattering properties. **b** The calculated transmission spectra of hole-array metasurfaces together with the normalised electric field profiles (|E|) at both resonances. The parameters of the design are *his* = 155 nm, *d*_1_ = 78 nm*, d*_2_ = 101 nm, *h*_*ITO*_ = 380 nm, and pitch Λ = 350 nm. **c** Coloured SEM image of the fabricated sample

A schematic illustration of the metasurface concept, design and fabricated samples is presented in Fig. 1. Figure 1a shows the geometrical design of the hole-array metasurface. Upon applying an electric potential to the contacts, the electric current flowing through the conductive indium tin oxide (ITO) strip on top of desired metasurface pixel converts the ITO ohmic resistance to heat. This heat induces a change in the silicon refractive index. It correspondingly leads to a resonance shift of the metasurface and an abrupt transparency change at the resonant wavelength^26–28^. The applied currents can be controlled by an electronic driving system. This way, programmable electrical switches can control individual pixels at a high modulation rate. Notably, the cooling effect rises from natural thermal conduction to the substrate and thermal radiation to the air.

Fig. 1b presents the simulated transmission spectra of the hole-array metasurface, consisting of holes with radii *d*_1_ = 78 and, *d*_2_ = 101 nm on a *h*_*is*_ = 155 nm thick amorphous silicon film, covered in *h*_*ITO*_ = 380 nm ITO. The insets images adjacent to the resonances depict the electric field profile (|E|), illustrating the strong contribution of the in-plane magnetic dipole (MD) for the resonance at a shorter wavelength (720 nm). The electric dipole (ED) is the dominant contributor to the scattering resonance at about 760 nm. the ED is mainly contributed by a toroidal dipole (TD) here. TD is an independent term induced by oscillating poloidal current in the multipole expansion of the electromagnetic field beside the magnetic and electric multipoles^29^. At Γ point, such a TD mode has zero overlaps between its mode profile and outgoing waves supported by our metasurface and thus belongs to the symmetry-protected bound state in the continuum (BIC) here^30–32^. Further analyses of the scattering contributions in spherical and Cartesian bases can be found in Supplementary Section 3. The symmetry of the lattice and structure geometries make the hole-array metasurface insensitive to the incident light polarisation direction. Therefore, at any polarisation direction, this design can exhibit an abrupt transmission modulation. While hole-array metasurface designs have been previously utilised for various applications^33–35^ the hole-array metasurface in this work enables us to exploit the connected film as a platform for uniform heating and temperature measurement by using a thermal camera (see Supplementary Section 4).

The design is subsequently implemented using standard electron-beam lithography and reactive ion etching, see Methods. The angled-view coloured scanning electron microscope (SEM) image in Fig. 1c shows the silicon hole-array membrane (in brown) that is covered by an ITO (blue) layer. The experimental characterisation of the fabricated sample is presented in Supplementary Section 5 and shows good qualitative agreement with our numerical simulations.

### Temporal response and dynamics of electrical tuning

**Fig. 2:**
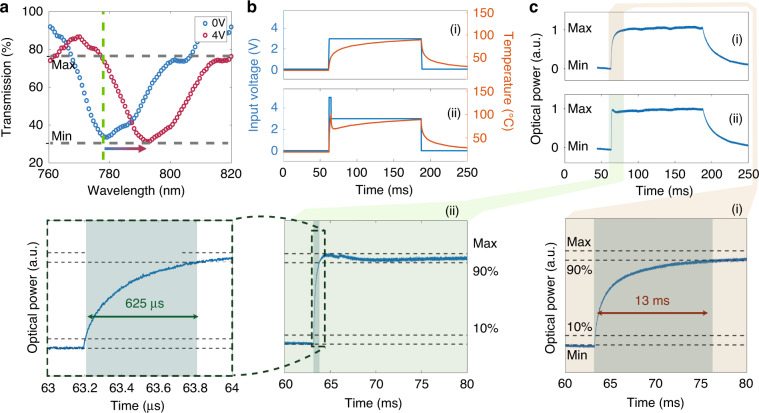
**Performance of the tuning method. a** Metasurface transmission spectra upon applying 4 V to its contact. **b** Simulation of ITO strip temperature rise by a square (i), or a modified asymmetrically descending step signal (ii). **c** Transmission optical power from a metasurface when a voltage is applied via (i) or (ii) manner. Magnified plots show the rise time under both bias profiles (i and ii) at the bottom

The thermo-optical response of a sample which exhibits a resonance at 780 nm at room temperature (blue) and after driving 4 V bias is shown in Fig. 2a. There is a good agreement in amplitude and wavelength of ED resonance between the experimental blue curve here, and the simulated curve in Fig.1, considering the difference wavelength windows shown in these figures. The slight shift of 20 nm is likely due to the difference between the ideal optical properties of materials (Si and ITO) in the theoretical calculations and experimentally deposited films. The metasurface temperature rise leads to a refractive index change of the resonators due to the thermo-optical effect^26^ and, subsequently, a shift in the resonance wavelength^26,27,36–38^. The transmission spectra of the sample, driven by different voltage biases, can be found in Supplementary Fig. S7. Through this geometrical design, we managed to convert the electrical switching to a localised flash heating and consequently to optical switching, all in a solid state. Figure 2b shows the simulation results of temperature rise versus the applied voltage profile where the width, length and thickness of the ITO heater, on the top of the silicon hole-array metasurface were 100 μm, 700 μm and 380 nm, respectively. The effect of ITO geometry on the temperature rise and the switching time is discussed in Supplementary Section 2. As can be seen in Fig. 2b(i), a voltage step change from 0 to 4 V leads to a gradual temperature change and, subsequently, a gradual change in the transmitted optical power, see Fig. 2c(i). As a result, it takes 13 ms for optical power to experience 10% to 90% variation. The optical switching performance and evaluation are described in the Materials and Methods section. Although 13 ms is still a competitive switching time in solid state switches, it is still longer than the video rate, therefore, not suitable for personal electronic devices equipped with displays.

To further decrease the optical switching time, we employed a step change with a short voltage spike <5 V. As can be seen in Fig. 2b(ii), such an asymmetric voltage application (blue curve) significantly shortens the temperature rise time (red curve). As an exciting outcome, the faster heating profile of the modified step voltage allows sub-millisecond switching time in the system. Fig. 2c(ii), and its magnified inset clearly demonstrate that within 625 μs, the measured optical power surges from 10% to 90%, far faster than the video frame rate. We have included an illustrative electronic circuit design in the supplementary information that can be used for designing such descending spike voltages (Supplementary Section 6). Also, we compared the optical response of metasurfaces activated by such input signals and square voltage (Supplementary Section 6). It is worth mentioning that utilising descending spiked voltage bias has not been demonstrated in other electro-optical systems, to date. The reason is the minimal effect of such a strategy on other optical systems. However, the geometry and physics of our switching approach enable employing spiked voltage bias in optics, for the first time. Such an innovative strategy paves the road for converting high-speed electrical switching to fast optical switching in solely solid-state devices.

**Fig. 3:**
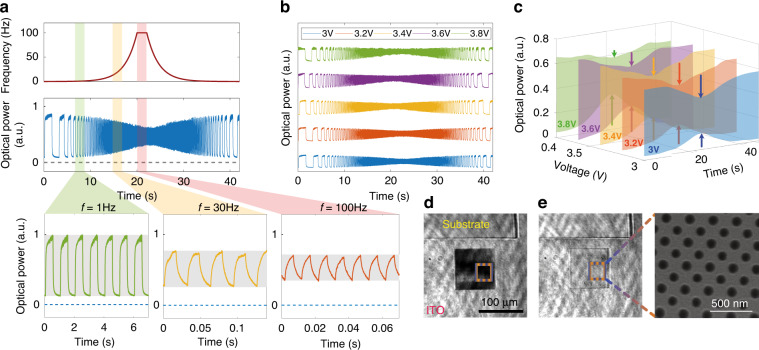
**Temporal modulation of the metasurface. a** Switching performance of the metasurface at different frequencies. The starting frequency is 0.25 Hz, reaching 100 Hz in 20 seconds on a logarithmic increment. Then the system operates with a 100 Hz switching time for 2 seconds and reverts to the 0.25 Hz in 20 seconds by a logarithmic decrement. The modulation performance of the metasurface is shown in the bottom plots at the frequency of 1 Hz (green), 30 Hz (yellow) and 100 Hz (pink). **b** Switching performance of the system at different bias voltages. **c** Lower and upper boundaries of the metasurface transmission intensity across the frequency sweep at different voltage biases. Optical images are taken from the metasurface **d** at room temperature and **e** when it is heated to 200 °C. The SEM image on the right-hand side shows the silicon membrane

Fig. 3 demonstrates the switching robustness, repeatability and tunability via this technique. In Fig. 3a, the metasurface switching frequency response is studied by logarithmic change of the voltage bias switching frequency. Initially, the switching frequency is increased from 0.25 to 100 Hz in the 20 s, and after locking it at 100 Hz for 2 s, it is reduced to 0.25 Hz in a reverse logarithmic manner in 20 s (with 3 V step profile voltage bias with a duty cycle of 0.5). Also plotted at the bottom of Fig. 3a are the switching responses of the metasurface at 1, 30 and 100 Hz. As can be seen, the relative transmission intensity can surge up to 9 times in each cycle (see bottom left panel). The measured intensity modulation depth at different frequencies can be seen in Supplementary Table S2. The system shows excellent reproducibility for the range of frequencies investigated. At higher switching frequencies (30–100 Hz), the optical responses’ minima and maxima increase and decrease gradually. To study this effect, the optical responses of the metasurface biased from 3 to 3.8 V voltage levels are plotted in Fig. 3b and 3c.

Fig. 3c shows the optical power for the investigated voltages, where the observed minimum (baseline) optical power levels increase while the maximum optical power levels decrease at higher frequencies. The response baseline increases due to the accumulated localised heat in the substrate and the microheaters, resulting in the resonance wavelength diverting from the laser wavelength. The system accumulates heat as the relaxation time at 0 V is shorter than the cooling time to room temperature (~68 ms). Consequently, the modulation depth of the system changes at different frequencies for different bias voltages (see Supplementary Table S2). In the demonstrated system, the cooling mechanism of the microheater in the system is limited to thermal radiation and convection air cooling. However, further active cooling approaches, such as liquid or thermoelectric cooling, can be used to improve the modulation depth and the system’s performance at higher frequencies. One more strategy for controlling heat accumulation can be utilising metasurfaces exhibiting resonances with higher quality factors. In such metasurfaces, the wavelength shift needed to alter the transmission intensity reduces, resulting in lower operating temperature and input voltage.

Fig. 3d shows the optical image of the metasurface where the minimum transmission intensity at room temperature matches the laser wavelength (780 nm). The metasurface is covered with an ITO strip, which acts as a microheater. Since the resonance wavelength of the metasurface and incident wavelength overlap, the metasurface forbids light transmission, looking opaque. Fig. 3e shows the metasurface transmission phase when the microheater heats it. The inset shows the SEM image of the silicon membrane, which was subsequently encapsulated by ITO. A video recording of the modulation with varied frequencies can be found in the Supplementary Information (Movie S1).

### Pixel-level programmable metasurface arrays

Lastly, we investigated the programmability of this type of tunable metasurfaces at a pixel level. Namely, we define four hole-array metasurfaces as individual pixels of a display. All four metasurfaces have the same parameters, however, can be independently tuned by a different microheaters for each pixel. Each metasurface pixel has a dimension of 100 × 200 µm, distanced by a 150 μm gap. This relatively large gap is important for reducing the thermal crosstalk between the pixels. However, it can be further reduced by fabricating small air grooves that are known to be extremely effective in thermal management^39^ As explained above, employing high-quality factor metasurfaces can also reduce the need for higher temperatures and, subsequently, more possibilities for active cooling and reducing the gap between the pixels. The four metasurfaces exhibit a resonance at 760 nm at room temperature. That resonance experiences a 20 nm red shift to 780 nm upon heating by the microheater with biasing voltage of 5 V. The spectral response of the metasurfaces is shown in Supplementary Fig. S8. To capture the metasurface optical response with our camera, we applied 5 V electrical pulses with 100 ms duration on an interval of 100 ms. The metasurfaces are illuminated by a broad laser beam at 780 nm wavelength and imaged onto a CCD camera. This slower tuning speed in this experiment is chosen due to the camera capturing speed.

**Fig. 4:**
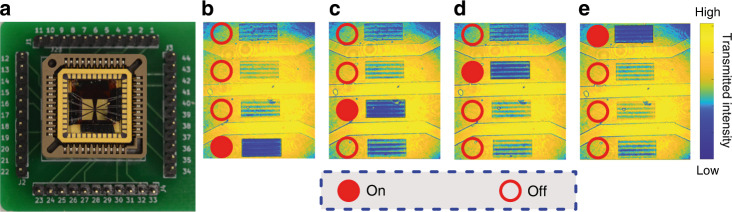
**The addressable metasurface pixels. a** Image of the metasurface mounted on a PCB for electrically programable pixels. **b**–**e** Images of the metasurface pixels driven by different microheaters at 780 nm wavelength

Fig. 4a shows a photograph of the meta-optical device connected to a printed circuit board (PCB) for electrical driving. Fig. 4b–e shows the images taken at different times when each pixel is turned on, as indicated by a solid circle on the left-hand side of the pixel. Upon switching the metasurfaces, their resonant wavelength is tuned to match the laser illumination wavelength. As a result, the spatial transmission of the driven pixel is suppressed by the resonance and the pixel appears dark in the camera. This example shows the metasurface modulation without noticeable crosstalk with the adjacent pixels. It is worth mentioning that heat accumulation may affect performance in extreme cases, such as long operation duration or high operating frequencies. The alleviating methods such as active cooling, lowering the operating temperature by using high-quality factor metasurfaces, or engraving micro-grooves between pixels can be helpful to minimise the potential thermal crosstalk in extreme conditions. The video captured from this switching is depicted in the Movie S2 of the SI. The fringes seen in the optical images of the metasurfaces are due to the EBL patterning imperfection, possibly due to issues with the tool’s pattern generator. However, they do not dramatically affect the performance of the device. We note that the sequence of the switching of the different pixels does not affect the performance, as the heat is dissipated into the thermally conductive sapphire substrate. Overall, this experiment demonstrates the programmable electrical control of spatially distributed metasurface pixels and opens opportunities for fast meta-optics displays.

## Discussion

In summary, we experimentally reported the first electrically tunable solid-state metasurface with a modulation depth of 90%, with an order of magnitude faster modulation rate than the video frame rate. By employing thermo-optical silicon hole array metasurfaces controlled by electrically driven localised transparent heaters, we introduced an effective way to adapt fast electrical switching by an optical switch. By employing an asymmetric voltage spike, we demonstrated 10% to 90% optical modulation in 625 μs. The proposed mechanism fills the technological gap for tunable metasurfaces capable of switching light effectively in the transmission regime at high frequencies. This is to note that the scope of this letter is to report this innovative technique rather than optimising the device. We believe incorporating an active cooling mechanism in the system is expected to enhance further the modulation depth and rate beyond the demonstrated values.

## Materials and methods

### Fabrication

A low-stress a-Si was deposited on a quartz substrate at 200 °C using the Plasma-enhanced chemical vapour deposition. An array of holes resembling the reverse design metasurface was exposed on the ZEP520A electro-resist by electron-beam lithography. After developing the resist, the resist was then used as an etching mask to transfer the hole pattern into the a-Si film by a dry etching process. Subsequently, ZEP520A electro-resist was also used for patterning the ITO layer as the transparent microheater. The ITO layer was deposited on the sample at room temperature by sputter deposition, and patterns were formed on the metasurfaces after the lift-off process. After annealing the sample at 300 °C, contact pads were lithographically fabricated with a 5 nm titanium layer as the adhesion layer and a 100 nm gold layer as the contact pads. A hole is drilled in the chip-carrier centre, enabling light-scattering measurements in the transmission regime. The device is then wire bonded to a chip carrier. The fringes observed in the optical images captured in Fig. 4 are due to the patterning imperfections that occurred during electron-beam lithography for the four-pixel samples.

### Electronic unit

The chip carrier socket was soldered to a printed circuit board and directly used for biasing the contacts by a power supply. A hole has been drilled through the chip carrier socket and the PCB board. A DC power supply was used for measurements that did not require a high switching speed, and an arbitrary function generator (Tektronix AFG3022B) was used for temporal measurements. A DAQ (USB-3114) was used for experiments that required multiple switchable power sources. In order to make sure the DAQ pins support adequate power into the system, multiple pins have been connected to the contact and programmed to supply the required voltage to the contacts simultaneously.

### Optical setup

A simple imaging setup depicted in Supplementary Fig. S9 was used for recording the images and the transmission of optical power measurements. A laser diode with a 780 nm illumination wavelength is collimated and used for illuminating the sample. Then the laser wavelength is tuned to the desired value by controlling the laser diode temperature. The rotatory disk usage was optional and only acted as a diffuser to reduce the speckle noise and fringes on the camera sensor. The laser beam passes through the sample and was collected by a ×20 objective. Then a beam splitter samples the output beam, and an infinity-corrected camera and a photodiode capture the sample image and its temporal optical response. For measuring the system’s temporal response, a ×10 objective is placed in front of the sample to focus light on the metasurface.

### Thermal camera calibration

A FLIR Lepton 3.5 camera installed on a PureThermal 2 Smart I/O Module operating in low gain mode is used for capturing the thermal imaging of the sample. A ZnSe lens with 15 mm focal length is used for image magnification. The transmission coefficient of the lens has been found by measuring a surface temperature with and without the lens from a polyvinyl chloride (PVC) tape (emissivity = 0.91 to 0.93). The relative emissivity of the a-Si silicon is measured by comparing the intensity captured by pixels from the a-Si film and the PVC film. This information is used to calibrate the device’s temperature.

### Modulation strength

The modulation depth *η* is calculated by:

$$\eta = \frac{{\Delta {{{\mathrm{I}}}}}}{{{{{\mathrm{I}}}}_{{{{\mathrm{absolute}}}}\,{{{\mathrm{max}}}}}}} \times 100 = \frac{{{{{\mathrm{I}}}}_{{{{\mathrm{max}}}}} - {{{\mathrm{I}}}}_{{{{\mathrm{min}}}}}}}{{{{{\mathrm{I}}}}_{{{{\mathrm{absolute}}}}\,{{{\mathrm{max}}}}}}} \times 100$$

where I_min_ and I_max_ are the minimum and maximum metasurfaces transmission intensity output. I_absolute max_ is the maximum achievable transmission output that can be measured at low switching frequencies. Based on Fig. 2c, the modulation depths of the metasurfaces at different voltages and switching frequencies are given in the Supplementary Table S2. The maximum modulation depth is achieved by applying 3.6 V to the system at low switching frequencies, at this temperature, the resonance peak is completely shifted out of the illumination wavelength. At smaller switching voltages, the modulation cannot reach its peak, and at larger voltages (i.e. 3.8 V), the transmission intensity declines as other resonance modes in the metasurface suppress it. The minimum output intensity (I_min_) increases to a higher value at higher switching frequencies. Larger transmission intensity output at larger switching input voltages suggests the gradual heat accumulation in the system at higher switching frequencies, as the voltage off-time is smaller than the cooling time of the system. Similarly, the I_max_ declines at high frequencies as the voltage on-time becomes shorter than the rise-time of the system. The output intensity of the system with larger input voltage amplitude at high frequencies is larger compared to when the system is derived at smaller voltage inputs, as larger voltages result in higher temperatures.

## Supplementary information

SI

Movie S1Please add the link of the file

Movie S2Please add the link of the file
