# Supplementary material for: Electrically programmable solid-state metasurfaces via flash localised heating
Source: Light Sci Appl. 2023 Feb 22;12:40. doi: 10.1038/s41377-023-01078-6 (PMC9944259; doi:10.1038/s41377-023-01078-6)
Supplement: Supplementary file 1 — SI [file 41377_2023_1078_MOESM1_ESM.docx]

Electrically Programmable Solid-State Metasurfaces via Flash Localised Heating

Khosro Zangeneh Kamali,^1^ Lei Xu,^2^ Nikita Gagrani,^1^ Hoe Tan,^1^ Chennupati Jagadish,^1^ Andrey Miroshnichenko,^3^ Dragomir Neshev,^1∗^ Mohsen Rahmani^2∗^.

Correspondence to: [dragomir.neshev@anu.edu.edu](mailto:dragomir.neshev@anu.edu.edu) and [mohsen.rahmani@ntu.ac.uk](mailto:mohsen.rahmani@ntu.ac.uk)

Supplementary Section 1 | Electrically Tunable Metasurfaces

Here we explain the shortcomings of major metasurface electrical tuning methods in details.

- Liquid crystals:

Liquid crystals are one of the widely used materials for tuning metasurface response by changing their surrounding medium refractive index. Liquid crystals, as indicated by their name, are not solid-state and are not favoured for some applications. Aside from not being solid-state, the liquid crystals switching speed are intrinsically limited because of the relatively slow process of Fréedericksz transition. Polarisation dependency is another liquid crystal tuning disadvantage that brings additional limitations to the acceptable input illumination polarisations. Since heat can act as another stimulus to alter the refractive index of liquid crystals, this method might not be robust enough in some applications.

- Carrier injection:

The scattering properties of metasurfaces can be altered by controlling their carrier concentration. Charge carrier density can be electrically modified in a component surface by electrostatic gating in a parallel plate capacitor configuration. Modulation via carrier injection often exhibits ultra-fast response. When it comes to shortcomings, one can name its relatively small modulation depth and work in the reflection regime. Moreover, this modulation method mostly operates in wavelengths longer than the visible (near-infrared and infrared light).

- Phase-change materials:

Using phase-change materials (PCMs) are widely used to introduce tunability. One of the shortcomings of the phase change materials is that reverting the phase from crystalline state to amorphous requires high temperature. This high temperature (> 600 ºC) disabled this type of tunable metasurfaces from being easily integrated with the CMOS devices, as high temperature degrades the p-n quality junctions and results in high noise read. Meanwhile, the heaters used for this type of material are often metallic, and the metasurfaces can only operate in the reflection regime. $\mathrm{VO}_{2}$ require much smaller temperature for material phase transition, though its phase transition from crystalline to amorphous is inherently slow.

- Pockels effect:

The materials that lack inversion symmetry experience refractive index change upon applying an electric field. This (Pockels effect) is used for tuning the metasurfaces surrounding environment and, therefore, their responses. Of the main shortcomings of this tuning method, one can mention that it can only be achieved for materials that lack the inversion symmetry, and this response is usually minimal. A comparison between different metasurface modulation methods can be found in Table S1.

Table S1. Techniques used for electrically tuning metasurfaces

| **Method** | **Phase Contrast** | **Intensity Contrast/ Modulation depth** | **Switching Speed** | **Direction** | **Stimulus** | **Demonstrated Wavelength** |
| --- | --- | --- | --- | --- | --- | --- |
| CI ^1^ | 360 | 3.5 to 4 % | 5.4 MHz | Reflection | -4 to 4 V | 1550 nm |
| CI ^2^ | 303 | 12 to 30 % | NA | Reflection | -6.5 to 6.5 V | 917 nm |
| CI ^3^ | 70 | 11 to 30 % | NA | Reflection | 0 to 10 V | 1552 nm |
| CI ^4^ | 270 | 0 to 16% | NA | Reflection | -6 to 6 V | ~8 um |
| CI ^5^ | 230 | 1.5 to 12% | NA | Reflection | -80 to 90 V | ~2.2 um |
| LC ^6^ | NA | NA | 1 kHz | Reflection | 0 to 30 V | 633 nm |
| PE ^7^ | NA | η = 37 % | 50 MHz | Transmission  and Reflection | -80 to 80 V | 1200 nm |
| PE ^8^ | NA | η = 67 % | 3 GHz | Transmission  and Reflection | -100 to 100 V | 1550 nm |
| PCM ^9^ | 360 | η ~ 80 % | kHz | Reflection | 0 to 5 V | 1640 nm |
| PCM ^10^ | NA | 4.3 to 14.5% | 200 ns | Reflection | 15 mA | 700 nm |
| PCM ^11^ | NA | ~ 30% | NA | Reflection | 0 to 11.3 V | ~1500 nm |
| PCM ^12^ | 250 | 23.5% | NA | Reflection | 0 to 13 V | ~1500 nm |
| This work | NA | η=90% | 100 Hz* | Transmission and Reflection | 0 to 5 V | 740 and 780 nm |

CI = Carrier Injection, LC = Liquid Crystal, PE = Pockels effect, PCM = Phase-Change Material
*can be increased by incorporating active cooling in the system

**Supplementary Section 2 | Heat Generation Modelling and Optimisation**

We calculate the generated heat by Joules heaters by:

$$P= \frac{V^{2}}{R}$$

where $P$ is the loss of the power (heat), $V$ is the applied voltage, and $R$ is the heating element resistance. The resistivity of the strip can be calculated by:

$$R= \rho\frac{l}{A}$$

where $A$ is the cross-sectional area, $\rho$ is the material's resistivity (ITO), and $l$ is the length of the slab. By considering Ohm's law ($V = I \times R$), the resistance of the heater and, therefore, the circuit current linearly increases with the width or height of the heater's strip. However, this heat is distributed in a larger volume of the conductive strip, which results in a constant temperature rise. Increasing the lengths of the strip increases its resistance, which reduces the heat generated in the system. Considering the increment in the volume of the heater, this results in heat generation being $l^{2}$ times smaller when the length of the strip is reduced.

To perform a more realistic calculation, we numerically calculated the system by COMSOL Multiphysics software, using “Joule Heating” interface (under Electromagnetic Heating Module). The heat transfer in the system is calculated by $\rho C_{p} \partial T/\partial t - \nabla. (k \nabla T)= Q_{e}$, where $\rho$ is the conductivity, $C_{p}$is the specific heat capacity, $k$ is the thermal conductivity, $\nabla T$ is the temperature gradient, and $Q_{e}$ is the source heat. In the resistive heating system, the heat source can be calculated by $Q_{e}=J . E$, where $J$ is the current density and the $E$ is electric field strength. The energy exit the system by radiation can be calculated by $q= \varepsilon\sigma(T_{amb}^{4}-T^{4})$, where $\varepsilon$ is the emissivity of the material, and the $\sigma$ is Stefan-Boltzmann constant. The cooling due to the convection is calculated by $q_{0}=h(T_{\mathrm{ext}}-T)$, where $h$ is the heat transfer coefficient.

In our simulations, we deployed the actual materials properties of the system components, cooling mechanisms such as air conduction and radiation cooling, and the heater's size and position on the substrate. Figure S1a shows the temperature of the ITO strip (width = 100 µm, length = 700 µm, thickness = 400 nm) after applying 5 V voltage bias for 250 ms. Figures S1b and S1c show the rise-time and temperature rise of the ITO strip, with different lengths, widths and thicknesses after applying 3 V to contacts at 250 ms. The trend shows that by increasing the ITO thickness and reducing the width and length of the strip, the maximum temperature increases. The rise time is shorter for ITO strips with short width and lengths, and no significant change was observed when the ITO thickness varies.


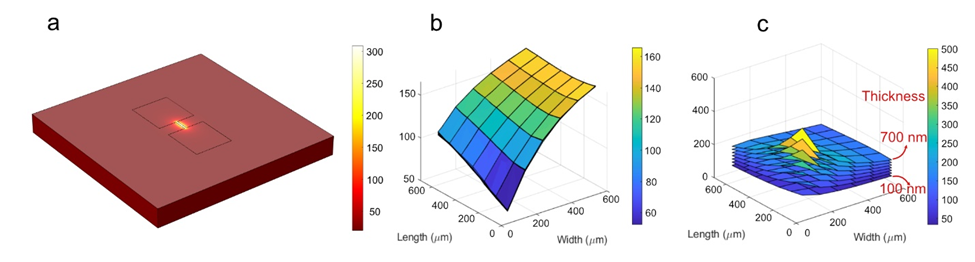


Fig. S1. Heat generation simulations by localised heaters. a, Thermal simulation of an ITO microheater (length = 700 µm, width = 100 µm, and thickness = 400 nm) after applying 5 V at 250 ms. The substrate is a 1 mm thick quartz and edge length of 10 mm. b, rise-time and c, the temperature of the ITO heater biased at 3 V, with different heater dimensions.

**Supplementary Section 3 | Modal analysis of the hole array metasurface**

Figure S2a shows the calculated multi-modal analysis of the hole-array metasurface, presented in spherical harmonics. The corresponding transmission spectrum is given in Fig. S2b. Figures S2c and S2d present the mode profiles at the two positions of both resonances at 710 nm and 750 nm, respectively.


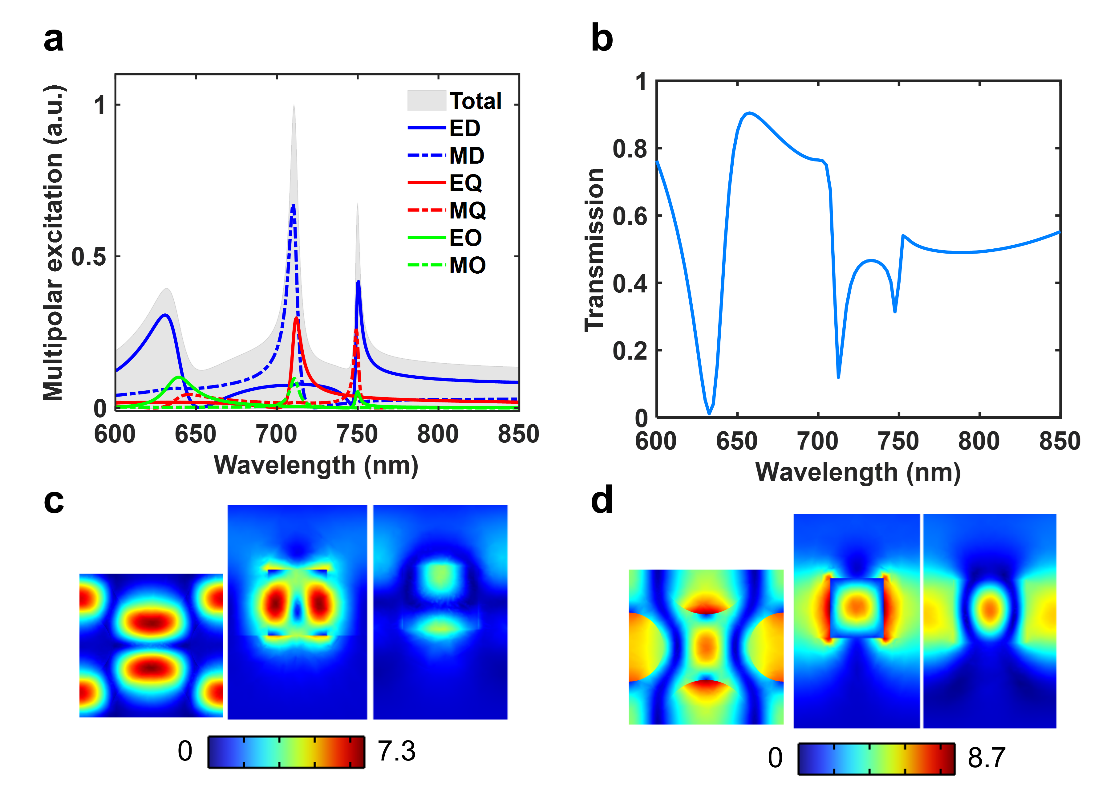


**Fig. S2. Metasurface multipolar analysis and electric near-field distribution.** **a**, Multipolar decomposition of the hole array metasurface. **b**, Transmission spectra of the metasurface. **c** and **d**, Electric near-field distributions for pump wavelength at 710 nm (**c**) and 750 nm (**d**), respectively.

By further performing the Cartesian electric multipolar analysis, we have shown that the resonance around 750 nm is indeed a symmetry-protected bound state in the continuum. A strong toroidal dipole (TD) mode is excited, as shown in Fig. S3a. The TD and MQ are bounded states due to the symmetry of our periodic system, and their mode profiles have zero overlap with the normal plane wave in our sub-diffractive system. Here, by controlling the difference of the two holes, we break the symmetry and excite the electric dipole moment $p_{y}$ serving as the leaky channel to couple the external light into the ideal bounded states TD and MQ, transferring the ideal bound state in the continuum (BIC) to quasi-BIC. It manifests itself as a sharp Fano feature in the transmission spectrum. We further fix the size of disk hole 1 and calculate the transmission spectrum with different sizes of disk hole 2, as shown in Fig. S3b. As can be seen, by tuning the difference of the two holes, the width of the resonances can be engineered. When the two holes have the same size, the resonance disappears, and the quasi-BIC is transformed into the ideal BIC, which cannot be excited by external normal plane-wave incidence anymore.^13,14^


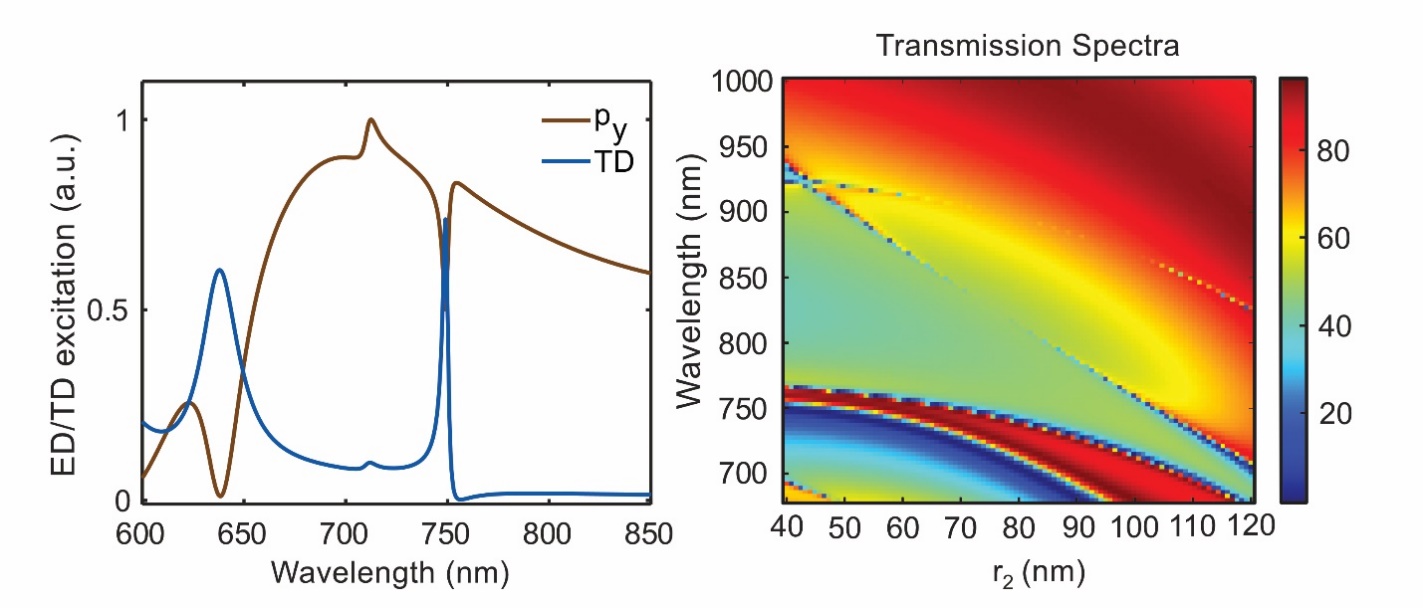


**Fig. S3**. **a**, Electric dipole and toroidal dipole resonance excitation. **b**, Transmission spectra of the metasurface with surveying the size of a hole type, while keeping the other home type size constant.

**Supplementary Section 4 | Temperature Build-up and the Metasurface Temporal Response**

The temperature build-up has been observed and recorded using a thermal camera in 100 Hz switching frequency, with different peak voltages from 3 to 3.8 V (duty cycle = 50%). This temperature build-up explains the increment in the baseline of Figs. 2b-c, as it causes a resonance shift in the metasurface, and causes the metasurface not to experience a full amplitude modulation.


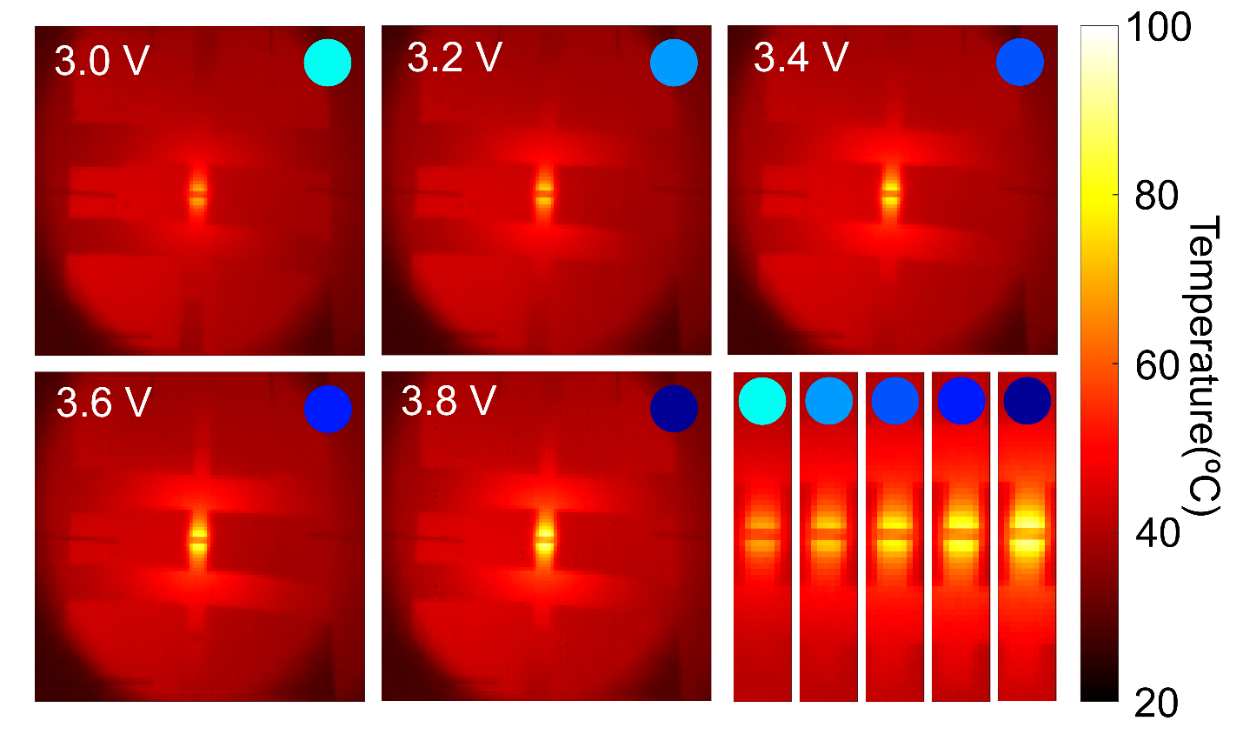


**Fig. S4. The effect of voltage on the build-up temperature at high frequencies.** The temperature build-up after continuously switching the metasurface for about 30 seconds with voltages from 3 to 3.8 V at 100 Hz.

Table S2. Modulation depth for a metasurface with different voltage bias at different frequencies.

| Voltage | 1 Hz | | | 30 Hz | | | 100 Hz | | |
| --- | --- | --- | --- | --- | --- | --- | --- | --- | --- |
|  | $\boldsymbol{I}_{\boldsymbol{min}}$ | $\boldsymbol{I}_{\boldsymbol{max}}$ | η (%) | $\boldsymbol{I}_{\boldsymbol{min}}$ | $\boldsymbol{I}_{\boldsymbol{max}}$ | η (%) | $\boldsymbol{I}_{\boldsymbol{min}}$ | $\boldsymbol{I}_{\boldsymbol{max}}$ | η (%) |
| 3 V | 0.0519 | 0.519 | 90 | 0.124 | 0.433 | 71.4 | 0.19 | 0.376 | 49.4 |
| 3.2 V | 0.0549 | 0.565 | 90 | 0.149 | 0.480 | 69 | 0.231 | 0.434 | 46.8 |
| 3.4 V | 0.0549 | 0.581 | 91 | 0.195 | 0.496 | 60.7 | 0.266 | 0.465 | 42.8 |
| 3.6 V | 0.0568 | 0.597 | 91 | 0.232 | 0.53 | 57.8 | 0.307 | 0.509 | 40.1 |
| 3.8 V | 0.0645 | 0.597 | 89 | 0.232 | 0.55 | 56.2 | 0.317 | 0.529 | 39.7 |


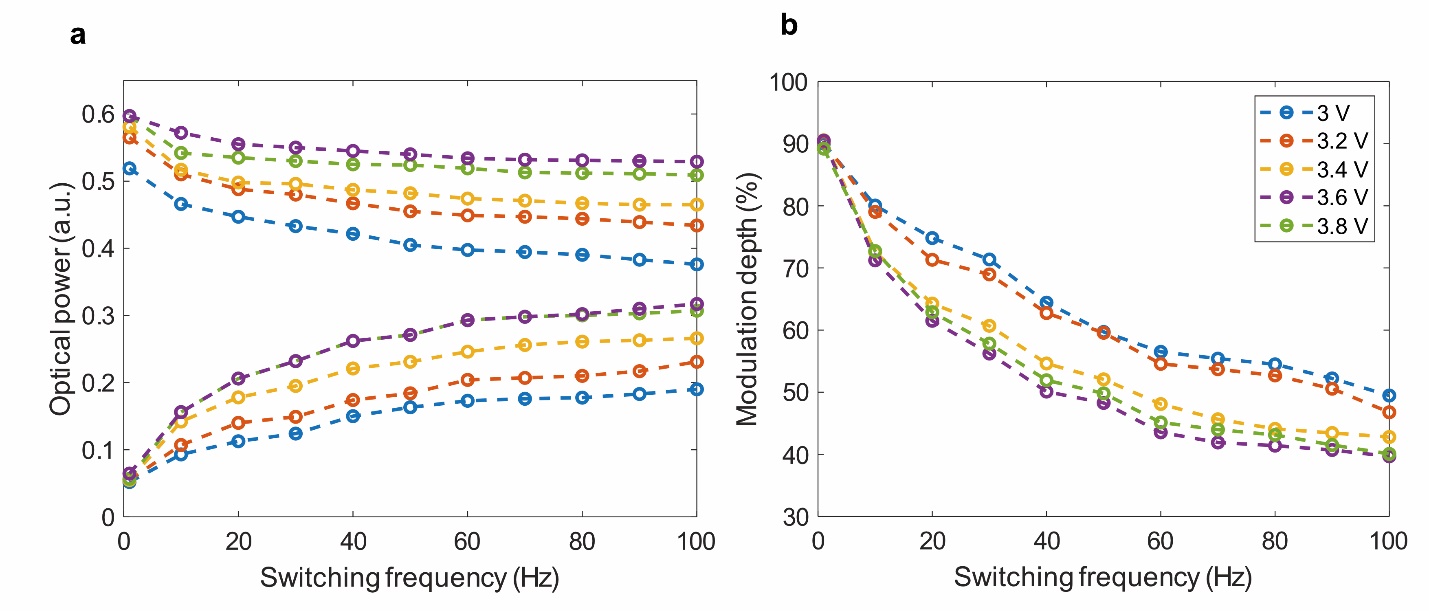


**Fig. S5. The switching response of the electrically tunable metasurface.** **a,** the observed optical power of the metasurface upon switching in different frequencies at different peak voltages by a photodetector. **b,** The calculated modulation depth of the metasurface is based on the data obtained in Fig. S5a.


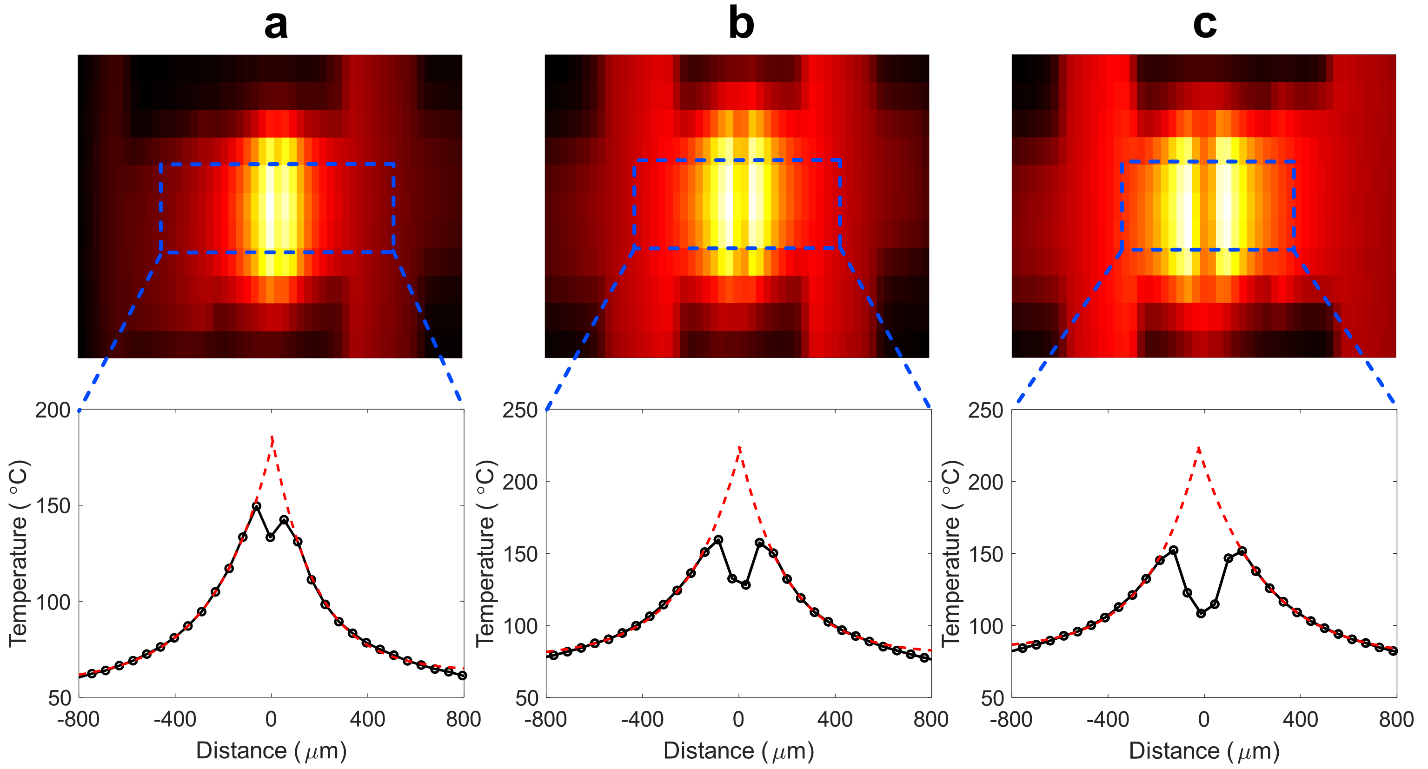


**Fig. S6. The system's temperature profile.** The temperature build-up after switching the microheaters for about 30 seconds, with widths **a**, 100 µm, **b**, 200 µm, and **c**, 300 µm.

**Supplementary Section 5 | Thermal Tuning of the Programmable Metasurface**

Two types of metasurfaces are presented in the manuscript that exhibit slightly different resonance wavelengths due to slight variations in their geometrical parameter. The spectral response of the metasurface shown for the temporal response measurements and the programmable metasurfaces are at different input voltages are depicted in Figs. S7 and S8, respectively.


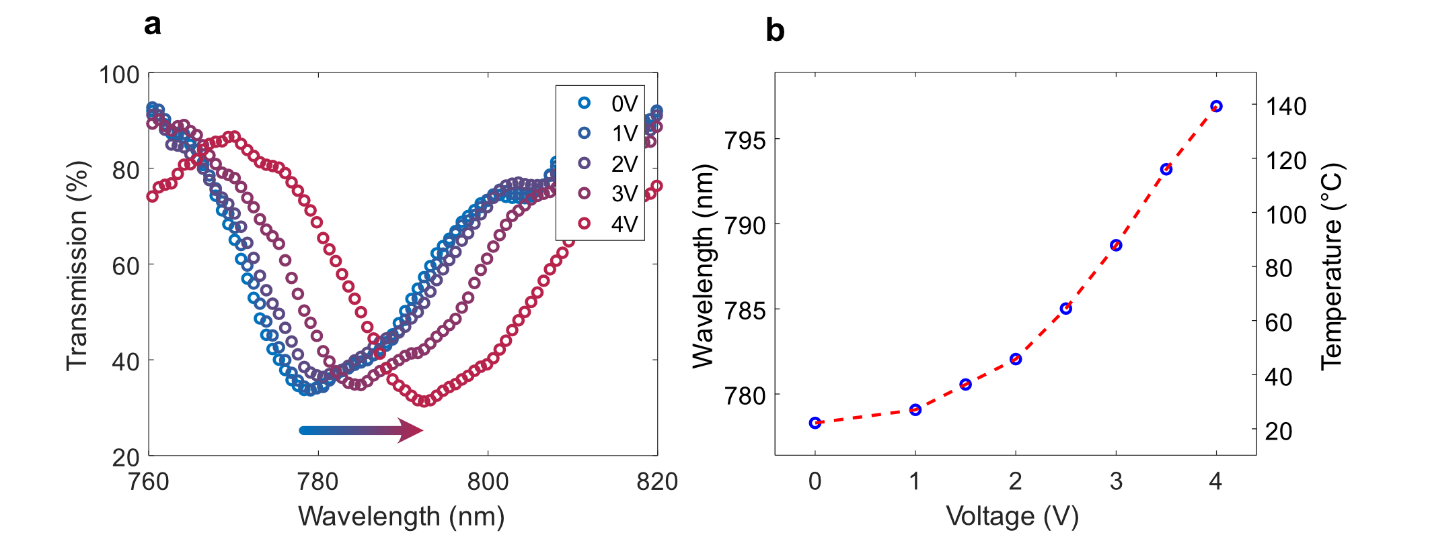
Fig. S7. The transmission spectra of the revered design metasurface at different bias voltages.

Experimental measurements of the transmission spectra of an individual metasurfaces at different DC bias voltages encapsulated in an ITO micro heater (width = 100 μm, length = 700 μm, and thickness = 380 nm).

Fig. S8. The transmission spectra of the four individually derived metasurfaces at different temperatures. Experimental transmission spectra of the four individual metasurfaces used as addressable optical switches from room temperature (blue) to ~200 ºC (orange).


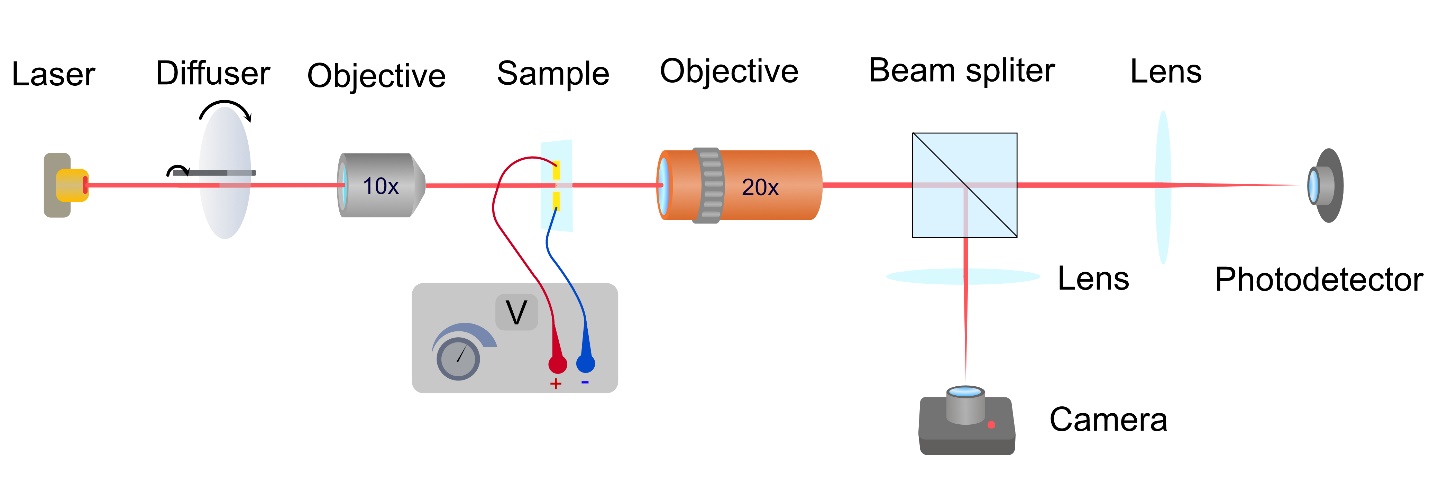


Fig. S9. The optical measurement setup. The optical setup used for metasurfaces imaging and temporal measurements.

**Supplementary Section 6 | Generation of descending spiked voltage and its performance.**

The schematics below show how voltage adder circuits enable descending spike voltage profile consisting of two voltage sources.

**Fig. S10. Voltage adder circuit schematics.** The voltage adder circuit schematics can generate simple asymmetrical voltages by adding two voltages. It consists of the part that adds up voltages (blue) and a simple amplifier.

The blue part is a basic voltage circuit that adds up the voltages from two separate power sources (signal generator). The output voltage of this part of the circuit is the average V_1_ and V_2_ in time. Upon demand, the orange circuit, which is an amplifier using op-amp component, can be used. The signal amplification is determined by $\frac{R5+R4}{R5}$. We simulated the generated voltage signals for this circuits using LTSpice software for 1, 10, and 100Hz frequencies. Figure S11 demonstrates the voltage profiles:

Fig. S11. Voltage adder circuit output. The voltage adder circuit output signal from a power source with an amplitude of 3 V (V_1_) and duty cycle of 50%, and a power supply with amplitude of 2 V and duration of 2.5 ms at 1 Hz (left), 10 Hz (middle), and 100 Hz (right).

Figure S12 shows the comparison between the optical response of metasurface activated by conventional square and descending voltage profile at different frequencies. The spike results in flow of more current in the circuit at the beginning of activation. As demonstrated, the effect of the spike voltage in reducing the modulation time is always significant. One can achieve a similar effect in such conditions by increasing the spike voltage and reducing its duration.

 
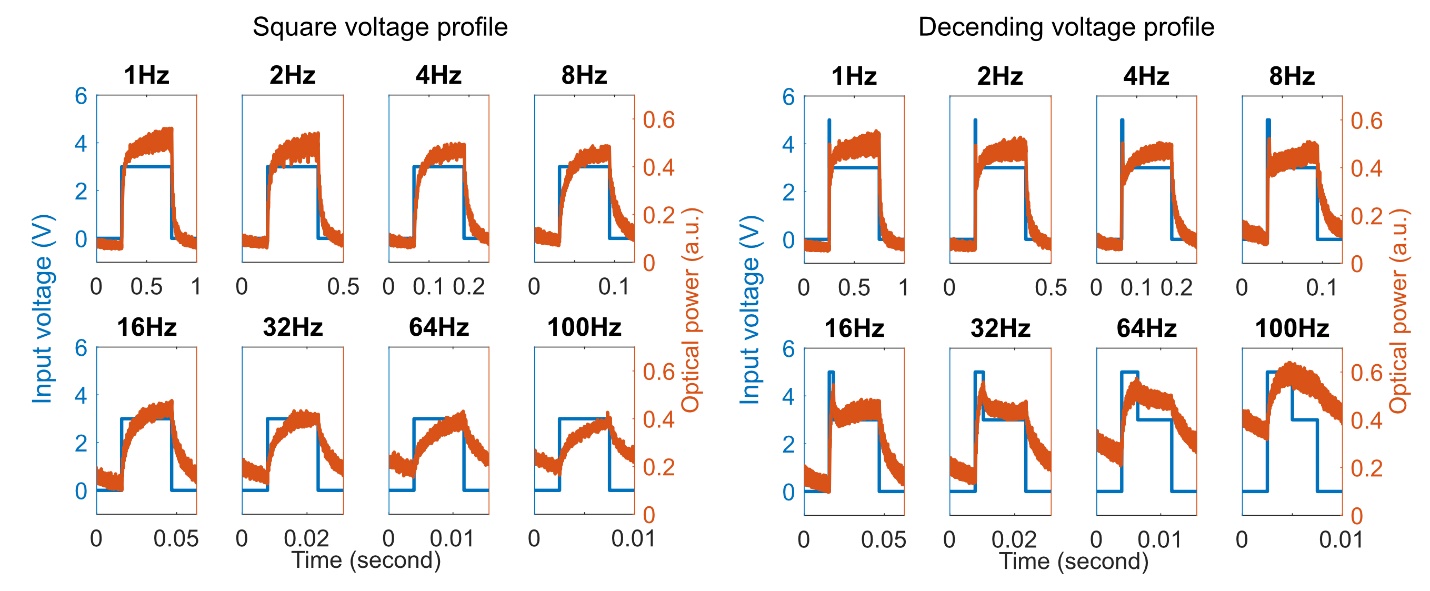


Fig. S12. Square and descending voltage operation at different frequencies. The optical response of the metasurface is activated by square and descending spike voltage at different frequencies.

Movie S1.

Movie S1 demonstrates the optical response of the metasurface described in Fig. 3. The electrical switching increased logarithmically from 0.25 Hz to 100 Hz in 20 s, and after 2 seconds of staying at 100 Hz, it is reduced to 0.25 Hz in a logarithmic reverse manner in 20 s.

Movie S2.

Movie S2 demonstrates the programmability of the system made of four individual metasurface in Fig. 4, controlled by four microheaters.

**References:**

1 Shirmanesh, G. K. *et al*. Dual-Gated Active Metasurface at 1550 nm with Wide (>300°) Phase Tunability. *Nano Lett* 2018; **18**: 2957–2963.

2 Wu, P. C. *et al.* Dynamic beam steering with all-dielectric electro-optic III–V multiple-quantum-well metasurfaces. *Nat Commun* 2019; **10**: 3654.

3 Shirmanesh, G. K. *et al*. Electro-optically Tunable Multifunctional Metasurfaces. *ACS Nano* 2020; **14**: 6912–6920.

4 Sherrott, M. C. *et al.* Experimental Demonstration of >230° Phase Modulation in Gate-Tunable Graphene-Gold Reconfigurable Mid-Infrared Metasurfaces. *Nano Lett* 2017; **17**: 3027–3034.

5 Karst, J. *et al.* Electrically switchable metallic polymer nanoantennas. *Science (1979)* 2021; **374**: 612–616.

6 Li, J. *et al*. Electrically-controlled digital metasurface device for light projection displays. *Nat Commun* 2020; **11**: 3574.

7 Benea-Chelmus, I. C. *et al*. Electro-optic spatial light modulator from an engineered organic layer. *Nat Commun* 2021; **12**: 5928.

8 Benea-Chelmus I. C. *et al.* Gigahertz free-space electro-optic modulators based on Mie resonances. *Nat Commun* 2022; **13**. doi:10.1038/s41467-022-30451-z.

9 Abdollahramezani, S. *et al.* Electrically driven reprogrammable phase-change metasurface reaching 80% efficiency. *Nat Commun* 2022; **13**: 1696.

10 Wang, Y. *et al.* Electrical tuning of phase-change antennas and metasurfaces. *Nat Nanotechnol* 2021; **16**: 667–672.

11 Zhang, Y. *et al.* Electrically reconfigurable non-volatile metasurface using low-loss optical phase-change material. *Nat Nanotechnol* 2021; **16**: 661–666.

12 Kim, Y. *et al.* Phase Modulation with Electrically Tunable Vanadium Dioxide Phase-Change Metasurfaces. *Nano Lett* 2019; **19**: 3961–3968.

13 Hsu, C. W. *et al*. Bound states in the continuum. *Nat Rev Mater* 2016; **1**: 16048.

14 Xu, L. *et al.* Enhanced four-wave mixing from multi-resonant silicon dimer-hole membrane metasurfaces. *New J Phys* 2022; **24**. doi:10.1088/1367-2630/ac55b2.
